# Trace Amine Associated Receptor 1 (TAAR1) Modulation of Food Reward

**DOI:** 10.3389/fphar.2018.00129

**Published:** 2018-02-27

**Authors:** Catherine F. Moore, Valentina Sabino, Pietro Cottone

**Affiliations:** ^1^Laboratory of Addictive Disorders, Departments of Pharmacology and Psychiatry, Boston University School of Medicine, Boston, MA, United States; ^2^The Graduate Program for Neuroscience, Boston University School of Medicine, Boston, MA, United States

**Keywords:** binge eating, addiction, compulsive, prefrontal cortex, inhibitory control

## Abstract

Eating disorders and some forms of obesity are characterized by addictive-like, compulsive eating behavior which contains numerous similarities with compulsive drug use. Food intake is in part mediated by reward and reinforcement processes that can become dysregulated in these disorders. Additionally, impairments in inhibitory control regulation of reward-related responding can cause or further exacerbate binge and compulsive eating. Dysfunctions in two neurotransmitter systems in the mesocorticolimbic pathway, dopamine and glutamate, are thought to contribute to maladaptive eating behaviors. The trace amine associated receptor 1 (TAAR1) system is a promising therapeutic target for compulsive eating behavior due to the role of TAAR1 in synaptic transmission and in the modulation of dopaminergic and glutamatergic signaling. In support of this notion, the TAAR1 agonist RO5256390 was found to decrease the reinforcing effects of palatable food-cues and to reduce binge-like and compulsive-like eating of palatable food. Additionally, prolonged, intermittent access to palatable food was shown to downregulate TAAR1 in the prefrontal cortex, suggesting a potential role for TAAR1 signaling in inhibitory control processes. Research into the role of TAAR1 in addiction, including TAAR1’s ability to modulate psychostimulant reward and reinforcement, bolsters support for TAAR1 agonism as a pharmacological treatment for compulsive eating and other addictive behaviors. This review summarizes the evidence for TAAR1 agonism as a promising therapeutic for compulsive eating behavior as well as the hypothesized mechanism responsible for these effects.

## Introduction

Eating disorders, including binge eating disorder (BED), bulimia nervosa (BN), anorexia nervosa (AN), ‘food addiction,’ and some forms of obesity are associated with significant economic and social costs (Lehnert et al., 2013; Whiteford et al., 2013) and limited effective treatments are available (Flament et al., 2012). A main driver of eating disorders is thought to be the increased availability of highly palatable and highly reinforcing food, rich in fats and sugars, which results in addictive-like overconsumption (Morris et al., 2015). Dysfunctions in dopaminergic and glutamatergic neurocircuitries that mediate reward/reinforcement and inhibitory control processes are hypothesized to drive binge and compulsive eating behaviors (Moore et al., 2017a). The trace amine-associated receptor 1 (TAAR1) system has recently gained attention as a neuromodulator of dopaminergic and glutamatergic signaling, making it a very promising therapeutic target for addiction-like disorders. To date, while a multitude of research has investigated the role of TAAR1 in drug addiction (Thorn et al., 2014; Harkness et al., 2015; Pei et al., 2015; Liu et al., 2016), very little is known about the modulatory role of TAAR1 in the rewarding properties of food. The scope of this mini-review is to discuss published studies pertaining to TAAR1 in relation to food reward.

## TAAR1 Agonism Blocks Binge Eating of Palatable Food

Binge eating is defined as consuming large quantities of food in a short period of time accompanied by a loss of control over food intake (American Psychiatric Association, 2013; Davis, 2013b). In a typical binge eating episode, palatable food, usually rich in fats and sugars, is consumed at a very high eating rate. Binge eating is a central feature of BED, BN, and some forms of AN, and is also highly prevalent in obese individuals (Dingemans and van Furth, 2012; Davis, 2013a). Binge eating is driven largely by hedonic reward mechanisms and results in similar neurochemical consequences as drugs of abuse (Rada et al., 2005; Avena et al., 2008; Cottone et al., 2008; Corwin et al., 2011). Thus, the ability of TAAR1 agonism to reduce psychostimulant reward and to modulate the mesolimbic dopaminergic signaling (Miller et al., 2005; Xie et al., 2007; Espinoza et al., 2015) led to its investigation as a potential therapeutic to decrease binge-like eating of palatable food intake in rodents (Ferragud et al., 2017). The effects of TAAR1 agonism were investigated in a rodent model of binge-like eating induced by limiting access to a highly palatable, sugary diet for 1 h/day (Ferragud et al., 2017), a paradigm which induces both binge eating and heightened eating rate in rodents. In this study, the selective TAAR1 agonist RO5256390 dose-dependently blocked binge-like eating of the palatable diet. The highest dose of 10 mg/kg was sufficient to reduce intake of the palatable diet to equivalent levels of standard chow intake in control subjects, which notably was not affected by the drug treatment. Interestingly, selective drug effects on palatable food intake were also observed when the doses of 1 and 3 mg/kg were injected. Furthermore, treatment with RO5256390 decreased the feeding rate for the highly palatable food.

In a different study, RO5256390 was also able to decrease, as well as slow down, responding for chocolate flavored pellets in rats (Pei et al., 2014). In this study, only the highest dose of RO5256390 (10 mg/kg) was able to affect responding for the chocolate pellets, while treatment with 3 mg/kg of RO5256390 did not reduce food responding. Therefore, the two mentioned studies reported dissimilar sensitivity of the drug effect at the different doses tested, which is likely attributable to differences in experimental design: Pei et al. (2014) capped daily intake at 40 pellets per session, while Ferragud et al. (2017) had no limit in responses, allowing for a more sensitive measurement of TAAR1 effects even at lower doses.

Another study, performed in mice, reported similar effects of TAAR1 agonism. R5166017 (0.3 mg/kg) was able to reduce high fat diet intake in a model of diet-induced obesity (Raab et al., 2016). Following a 10-h fast, acute injection of the TAAR1 agonist reduced the total number of meals and duration of meals during the first hour compared to obese mice injected with vehicle as well as TAAR1 knockout (KO) mice (Raab et al., 2016). Additionally, subchronic treatment with the TAAR1 agonist (admixed in the diet to deliver an approximate dose of 3.5 mg/kg) reduced daily intake of the high fat diet by 25% and reduced body weight by 6.6% in obese mice (Raab et al., 2016).

**FIGURE 1:**
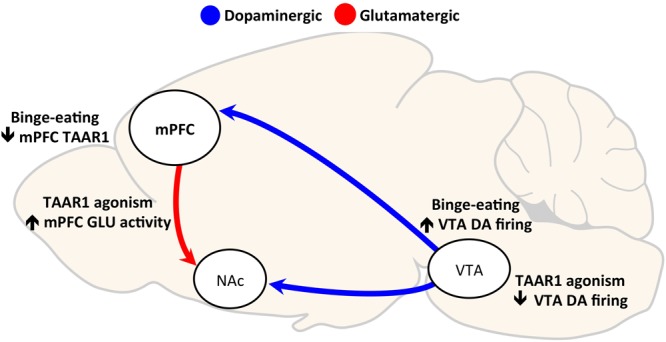
Hypothesized mechanisms of Trace Amine Associated Receptor 1 (TAAR1) agonism effects on compulsive eating. Two hypothesized mechanisms by which TAAR1 agonism may reduce binge eating are through: (1) decreasing VTA neuronal firing, and (2) increasing glutamatergic activity in the mPFC. Binge eating of palatable food repeatedly activates the VTA to NAc dopaminergic reward pathway (Rada et al., 2005). TAAR1 agonism decreases VTA neuron firing through activation of inwardly rectifying K^+^ channels (Revel et al., 2011, 2013); thus, its effects on reducing binge eating may be mediated by preventing hyperdopaminergic activity. Secondly, prolonged, intermittent binge eating of palatable food is a decrease in TAAR1 protein expression in the medial prefrontal cortex (mPFC) (Ferragud et al., 2017). The loss of TAAR1 has been shown to cause hypoglutamatergic function in mPFC (Espinoza et al., 2015), and low PFC activity is correlated with loss of control over eating (Balodis et al., 2013). Therefore, we hypothesize that TAAR1 agonism reduces binge, compulsive eating in part through restoring impaired mPFC activity [DA, dopamine; GLU, glutamate; mPFC, medial prefrontal cortex; NAc, nucleus accumbens; TAAR1, trace amine-associated receptor 1; VTA, ventral tegmental area].

Interestingly, partial agonism at TAAR1 (by RO5203648 or RO5263397) has been shown to have no effects on food self-administration (Pei et al., 2014) or to enhance responding for food under a progressive ratio schedule (Pei et al., 2014, 2017). Selective TAAR1 full agonists are known to decrease the frequency of dopamine neuron firing in the VTA through activation of inwardly rectifying K^+^ channels (Revel et al., 2011). However, partial TAAR1 agonism (Revel et al., 2012) as well as TAAR1 antagonism (Bradaia et al., 2009) have opposite effects, suggesting that TAAR1 maintains a degree of baseline or constitutive activity. Thus, while the divergence in effects on food reward between the full and partial TAAR1 agonism is not fully elucidated, we hypothesize that TAAR1 full agonist effect on binge eating lies in its ability to reduce VTA dopaminergic activity (see **Figure 1**).

## TAAR1 Agonism Reduces the Reinforcing Properties of Palatable Food-Associated Cues

Environmental food-associated stimuli exert a powerful control over behavior and can robustly enhance the desire to binge, even in satiated humans (Ng and Davis, 2013; Boswell and Kober, 2016) and animals (Everitt and Robbins, 2005; Giuliano and Cottone, 2015; Robinson et al., 2015). There is evidence in binge eating humans and animals of increased sensitization to palatable food cues (Cowdrey et al., 2011; Dimitropoulos et al., 2012; Lawrence et al., 2012; Meule et al., 2012; Schmitz et al., 2014; Hendrikse et al., 2015; Shank et al., 2015; Smith et al., 2015; Velazquez-Sanchez et al., 2015). In humans, measures of increased sensitivity are found to be associated with increased food seeking and eating (Lawrence et al., 2012), body mass index (Shank et al., 2015), and severity of self-reported binge eating symptoms (Schmitz et al., 2014).

A procedure used in the preclinical laboratory setting to assess the magnitude of influence of a conditioned stimulus (CS) over food or drug-seeking behavior is the second-order schedule of reinforcement (Giuliano et al., 2012; Giuliano and Cottone, 2015). In this paradigm, a stimulus is paired with palatable food (or another primary reinforcer) and is able to enhance or maintain responding, even in the absence of the primary reinforcer (Everitt and Robbins, 2000). In a second-order schedule of reinforcement, sessions typically comprise of consecutive intervals with the first one occurring before presentation of the palatable food and allowing the evaluation of the conditioned reinforcing properties of cues in absence of reinforcer (Giuliano and Cottone, 2015; Velazquez-Sanchez et al., 2015). In animals with a history of binge-eating a highly palatable, sugary diet, conditioned reinforcers elicit and maintain vigorous food seeking behavior as measured in a second-order schedule of reinforcement (Smith et al., 2015; Velazquez-Sanchez et al., 2015). The TAAR1 full agonist, RO5256390, was shown to block food-seeking behavior under this schedule, bringing responding to control levels (Ferragud et al., 2017). Importantly, the drug’s effect on food seeking was observed during the first interval (before food ingestion occurred), suggesting an effect on the incentive value of palatable food. Furthermore, no effects were observed in animals without a history of binge eating that were responding for standard chow food.

Another paradigm used to test the rewarding properties of food or drugs and the ability of paired contextual cues to drive behavior is the conditioned place preference (CPP) test. In animals with a history of binge-eating a highly palatable, sugary diet, following conditioning, tactile and visual cues associated with the highly palatable food are able to induce a strong place preference, unlike stimuli associated to the standard chow diet (Velazquez-Sanchez et al., 2015; Ferragud et al., 2017). When given prior to the CPP test, RO5256390 fully and selectively blocked the expression of CPP (Ferragud et al., 2017).

These experiments show that TAAR1 agonism greatly reduced the strength of palatable-food associated stimuli in driving behavior in rats with a history of binge eating. Though exact mechanisms have not yet been examined specifically for palatable food-cues, studies have found that the ability of TAAR1 to decrease the ability of drug-paired cues to elicit cocaine-seeking behavior can be pinpointed the ventral tegmental area and the prelimbic region of the medial prefrontal cortex (mPFC) as critical sites of action for TAAR1 in reducing cue-induced seeking behavior and drug-related memory expression (Liu et al., 2016, 2017).

## TAAR1 Agonism Blocks Compulsive-Like Eating

Compulsivity is a behavioral construct observed in different disorders, including drug addiction and disorders of pathological eating, where there is a loss of control over behavior that results in continued use despite incurring negative consequences (e.g., medical, psychological, emotional, and social impairment) (Cottone et al., 2012; American Psychiatric Association, 2013; Rossetti et al., 2014; Velazquez-Sanchez et al., 2014; Smith et al., 2015; Moore et al., 2017c). Compulsive eating behavior is characteristic of certain forms of obesity, BED, BN, certain forms of AN, and food addiction (de Zwaan, 2001; Davis and Carter, 2009; Gearhardt et al., 2009; American Psychiatric Association, 2013; Davis, 2013b; Volkow et al., 2013; Hone-Blanchet and Fecteau, 2014). Compulsive eating behavior has been dissected into three functional domains: habitual overeating, eating to alleviate a negative emotional state, and overeating despite negative consequences (Moore et al., 2017a,b,c), engendered by maladaptive habit formation (Furlong et al., 2014), the emergence of a negative affect (Cottone et al., 2009; Blasio et al., 2013; Iemolo et al., 2013), and a failure of inhibitory control systems (Velazquez-Sanchez et al., 2015; Nieh et al., 2015; Myers, 2017), respectively. ‘Loss of control’ is thought to result from deficits in inhibitory control mechanisms responsible for the suppression of inappropriate actions. These deficits confer vulnerability to the addictive behavior and/or emerge from persistent and prolonged palatable food overconsumption (Volkow et al., 2013; Velazquez-Sanchez et al., 2014).

To test continued overeating despite consequences in animals, a commonly used paradigm is the light/dark conflict test, where feeding is typically suppressed in control rats when they face the aversive bright compartment of the light/dark box. However, rats with a history of intermittent access to palatable food will compulsively consume the palatable diet in face of the risky environment (Cottone et al., 2012; Dore et al., 2014; Velazquez-Sanchez et al., 2014; Smith et al., 2015). TAAR1 activation was shown to fully block compulsive eating of palatable food, reducing intake in the bright compartment by 75%, compared to vehicle treatment (Ferragud et al., 2017). Importantly, the study confidently excluded the alternative interpretation that drug’s effect was induced by a potential anxiogenic profile as RO5256390 exerted no effect on anxiety-like behavior in a defensive withdrawal test (Ferragud et al., 2017).

Compulsivity shares some commonalities with impulsivity in that both constructs involve a dysfunction in inhibitory control, mediated by frontal cortex projections to subcortical regions (Robbins et al., 2012). Dysfunctions in inhibitory control can manifest as impulsive-like behavior, are hypothesized to contribute to the emergence of compulsive drug use and compulsive eating (Belin et al., 2008; Dalley et al., 2011; Velazquez-Sanchez et al., 2014), and are thought to be caused or exacerbated by exposure to drugs of abuse and highly palatable food (Jentsch and Taylor, 1999; Vickers et al., 2017). TAAR1 has been linked to impulsive-like behavior, likely through modulation of prefrontal glutamatergic neurotransmission. Deletion of TAAR1 in mice reduced prefrontal glutamatergic activity, coupled with heightened impulsive-like behavior, determined as high premature and inter-trial interval responding in a Fixed Interval-Peak Interval test (Espinoza et al., 2015). While TAAR1 partial agonism had no effects in TAAR1 KO mice, control mice treated with RO5203648 displayed decreased premature correct responses in an FI-30 schedule of reinforcement task (Espinoza et al., 2015), a measure of impulsive-like behavior (Berger and Sagvolden, 1998). Thus, TAAR1 effects on impulsive-like behavior are hypothesized to be attributable to the modulation of the prefrontal glutamatergic signaling that is critical to inhibitory control processes and is dysregulated in addiction (van Huijstee and Mansvelder, 2014). Furthermore, in a primate model, TAAR1 agonism reduced impulsivity and increased cognitive flexibility (Revel et al., 2013), another component of executive function that is often impaired in addiction and in disorders of compulsivity (Banca et al., 2016). Thus, we hypothesize that TAAR1 effects on compulsive eating may be mediated in part through a restoration of deficits in inhibitory control functions through restoring prefrontocortical glutamate activity (see **Figure 1**).

## Binge-Like Eating Decreases TAAR1 Protein Expression in Prefrontal Cortices

Inhibitory control processes are largely governed by prefrontocortical circuits, and dysfunctions in this pathway are thought to underlie the loss of control and continued intake despite aversive consequences (Tomasi and Volkow, 2013; Volkow et al., 2013; Moore et al., 2017a,b,c). Decreased activity in the prefrontal cortex (PFC) is observed in obesity, and is associated with decreased inhibitory control (Volkow and Wise, 2005).

Trace Amine Associated Receptor 1 modulation of prefrontocortical activity is one hypothesized mechanism for the effects on compulsive eating and other addictive behaviors. TAAR1 protein expression was decreased in the mPFC of animals with a history of binge eating of palatable food, compared to controls fed a standard chow diet (Ferragud et al., 2017). As summarized above, binge-like eating rats also display compulsive-like eating behavior, which is fully blocked by pretreatment with the full agonist of TAAR1 RO5256390 (Ferragud et al., 2017). Therefore, based on these results, we hypothesize that the RO5256390 may be able to restore the loss of function in prefrontocortical TAAR1 transmission, which is responsible for the observed compulsive, binge eating (see **Figure 1**). In line with this hypothesis, TAAR1 KO mice display behaviors consistent with prefrontal dysfunctions, such as perseverative behavior and impulsivity, suggesting that a deficiency in TAAR1 may result in deficits in cognitive function and behavioral flexibility (Espinoza et al., 2015). Furthermore, intra-mPFC (specifically infralimbic, IL, but not prelimbic, PrL) administration of the TAAR1 agonist decreased binge-like eating without affecting the intake of regular chow (Ferragud et al., 2017). While TAAR1 mRNA expression is equivalent in the IL and PrL (Liu et al., 2017), the IL cortex is crucial for the development and expression of inflexible reward seeking as well as habitual behavior (Barker et al., 2014; Moorman et al., 2015). However, it is likely that other TAAR1-expressing brain areas contribute to the systemic effects of TAAR1 agonism, as the magnitude of food intake reduction following intra-IL RO5256390 microinfusion was lower than that observed following intraperitoneal administration (Ferragud et al., 2017). It is not yet known if TAAR1 expression patterns are altered in obesity or other paradigms of overeating, or if this effect is specific to binge eating behavior.

## Discussion and Conclusions

The rise of eating-related disorders, including some forms of obesity, BED, BN, AN, and food addiction has prompted much research, and has resulted in a few medications with varying levels of effectiveness (Reas and Grilo, 2014). However, many patients constantly relapse in symptoms that include binge and compulsive eating, failing to achieve significant health and psychological benefits (Ferguson and Spitzer, 1995; Reas and Grilo, 2014). As TAAR1 agonism has been shown to reduce various maladaptive eating behaviors, it can indeed be regarded as a very promising therapeutic target for compulsive eating behavior, even though much more research is needed at this point. Recently, Lisdexamfetamine (LDX) was approved for the treatment of BED, and has been shown to reduce binge eating behaviors as well as compulsive eating behaviors (assessed with the Yale-Brown Obsessive Compulsive Scale modified for Binge Eating; Y-BOCS-BE) (Hudson et al., 2017). Interestingly, TAAR1 is also activated by amphetamine (Borowsky et al., 2001), the active metabolite in LDX (Goodman, 2010). LDX and TAAR1 agonism may, therefore, work through similar mechanisms to restore dysfunctional signaling in prefrontal areas, alleviating impaired inhibitory behaviors.

Importantly, TAAR1 effects on compulsive, binge eating are being driven by direct actions on food reward mechanisms, rather than homeostatic needs. Evidence for this is twofold. The first is that no food restriction or deprivation was used in the above-summarized studies showing its efficacy (Pei et al., 2014; Ferragud et al., 2017). Second, in a control experiment in food deprived rats, TAAR1 agonism had no effect on food intake at doses which reduced palatable food binging (Ferragud et al., 2017). Thus, agonism of TAAR1 specifically disrupted responding for palatable food, but not standard chow (in either *ad libitum* fed or food-restricted rats).

Though TAAR1 agonism effects on binge, compulsive eating are not being mediated by homeostatic mechanisms, it is interesting to note that TAAR1 activation has been studied for its metabolic effects in models of obesity and diabetes (Raab et al., 2016). In the periphery, TAAR1 agonism has been shown to have incretin-like effects, reducing high-fat food intake and body weight in diet-induced obesity mice, as well as normalizing glucose levels and restoring insulin sensitivity in diabetic mice (Raab et al., 2016). Considering the high occurrence of compulsive eating behavior with obesity (Dingemans and van Furth, 2012; Davis, 2013a), and that obesity greatly increases risk for diabetes (World Health Organization, 2000), TAAR1 agonism may act to reduce the amount of food eaten, but also to alleviate the comorbid metabolic symptoms that result from overeating.

The TAAR1 full agonist RO5256390 was not found to have any effects on anxiety-like (Ferragud et al., 2017) or depressive-like behavior (Revel et al., 2013; Ferragud et al., 2017). TAAR1’s specific effects of treating compulsive eating behavior without necessarily having effects on mood or emotion, highlights the multifaceted and parallel processes that lead to behavioral expression of compulsive eating. It is important to note that investigation into TAAR1 effects on food addiction mechanisms is in its nascent stages, with the majority of evidence coming from a few studies. Much more research is needed, including assessment of clinical efficacy in heterogeneous human populations. The targeting of specific cognitive processes, such as food cue reinforcement and inhibitory control mechanisms, will hopefully lead to more effective treatment strategies for compulsive eating and other addictive behaviors.

## Author Contributions

All authors listed have made a substantial, direct and intellectual contribution to the work, and approved it for publication.

## Conflict of Interest Statement

The authors declare that the research was conducted in the absence of any commercial or financial relationships that could be construed as a potential conflict of interest.
